# Care Seeking Behavior of Chest Symptomatics: A Community Based Study Done in South India after the Implementation of the RNTCP

**DOI:** 10.1371/journal.pone.0012379

**Published:** 2010-09-20

**Authors:** Niruparani Charles, Beena Thomas, Basilea Watson, Raja Sakthivel M., Chandrasekeran V., Fraser Wares

**Affiliations:** 1 Tuberculosis Research Center, Indian Council of Medical Research, Chennai, India; 2 Office of the WHO Representative to India, New Delhi, India; National Institute for Infectious Diseases L. Spallanzani, Italy

## Abstract

**Introduction:**

With the creation of the Revised National TB Control Programme (RNTCP), tuberculosis services have become decentralized and more accessible. A 1997 study prior to RNTCP implementation reported that most chest symptomatics accessed first private health care facilities and a general dissatisfaction with government health facilities. The study was repeated post-RNTCP implementation to gain insight into the current care seeking behavior of chest symptomatics.

**Methodology:**

A cross-sectional community-based study carried out between March-August 2008 in 4 sites (2 rural [R] and 2 urban [U]) from the same two districts of Chennai and Madurai, southern India, as in the 1997 study. Six hundred and forty chest symptomatics were identified (R 314; U 326), and detailed interviews were done for 606 (R311; U295).

**Results:**

Prevalence of chest symptomatics in the urban and rural areas were 2.7% and 4.9% respectively (*p*<0.01), and was found to increase with age (Chi-square for trend, p<0.01). Longer delays in seeking care were seen amongst symptomatics above 45 years of age (*p* 0.01), and those who had taken previous TB treatment (*p = *0.05). Overall, 50% (222/444) of the chest symptomatics approached a government health care facility first (R 142 (61%); U 80 (38%), p = <0.001). This was significantly (*p*<0.001) more than were observed in the 1997 study, where only 38.4% approached a government facility first. Sixty two (28%) of the 222 made a second visit to a government facility (R26%; U31%), while 17% shifted to a private facility (R14%; U21%). Dissatisfaction with the health care facility was one of the major reasons expressed.

**Conclusions:**

It appears that the RNTCP has had an impact in the community with regard to the availability and accessibility of TB services in government health facilities. However the relatively high levels of subsequent shifting to private health facilities calls for urgent action to make government facilities more patients friendly with quality care facilities in the delivery of RNTCP services.

## Introduction

Tuberculosis control activities in India have made huge strides with the introduction of the Government of India's Revised National Tuberculosis Control Programme (RNTCP), which adopted and adapted the core elements of the internationally recommended Directly Observed Treatment, Short course (DOTS) strategy [1]. The RNTCP, has been implemented keeping patient's reach and care as its prime concern. It has been implemented in the whole of the southern Indian state of Tamilnadu since 2000, and services are decentralized, and within easy reach of the majority of the population. As RNTCP is based on “passive case finding”, chest symptomatics are encouraged to report to public health facilities for free investigations and subsequent treatment of TB. In 1997, prior to the implementation of the RNTCP, a community based study of the care seeking behavior of chest symptomatics from urban and rural areas of the southern Indian state of Tamilnadu was carried out [2]. Important findings of the study were that 30% of chest symptomatics did not seek care from any health care provider, and that private health care facilities were the first point of contact for 57% of urban and 48% of rural chest symptomatics who did take action. The major reasons given for this preference were proximity to residence and the patients' perception that quality care was available in private clinics. Dissatisfaction with government health facilities was also reported.

With the implementation of RNTCP across India since early 2006, it is important to understand the current care seeking behavior of chest symptomatics in the same districts as compared to the findings reported in 1997 [2]. In addition to measuring any changes in health seeking behavior, the study reported on here also sought to identify barriers in accessing treatment at government health facilities under RNTCP. The study findings will help in strengthening the RNTCP and in planning of appropriate advocacy activities on TB control both for the community and for the health personnel.

## Methods

### Ethics Statement

This activity was reviewed and approved by the institutional ethics committees of the Tuberculosis Research Centre, Chennai, Indian Council of Medical Research and written informed consent have been obtained from all the study participants.

### Setting

This community based study was conducted in 4 sites (2 rural and 2 urban) from the same two districts of Chennai and Madurai, southern India, as in the 1997 study. The sites were further broken down into divisions and wards.

### Sampling and data collection process

In order to compare the findings of the 1997 study conducted prior to RNTCP implementation, the same probability proportional to size method was used to select 30 divisions/30 wards in each of the urban sites (Chennai and Madurai) and rural (Melur and Poonamalle) communities. From each division or ward, as compared to the previous study the adjacent street to the previous street selected randomly was considered and finally a house was chosen randomly from the selected street. The interviewers contacted the head of the selected household or any responsible person and the purpose of the visit and an overview of the study were explained. If the respondent was willing to share information, details on the number of chest symptomatics in the household if any, were elicited after getting their written consent. Chest symptomatics were defined as persons with symptoms of a productive cough for 3 weeks or more, with or without chest pain, fever, and loss of weight or haemoptysis during the 3 months prior to the visit. If a symptomatic was identified, the interviewer asked for his/her willingness to participate in the study which involved an interview by the interviewer at a time and place convenient to him or her. If he/she was available and consented to be involved in the study and spare the time, the interview was done the same day. If the respondent was not available, the interviewer fixed a date and time for the interview based on the convenience of the respondent. A maximum of 3 attempts was made to meet the eligible respondent (chest symptomatic) if willing to be a part of the study.

The study aimed to enroll 600 symptomatics, 150 in each of the four sites (2 in Chennai and 2 in Madurai).

### Data collection

A semi-structured interview schedule was used to elicit information. The interviews were done by trained investigators after obtaining written consent from participants prior to the interview. The schedule covered information on socio-demographics, knowledge of TB, health care seeking behavior, time of care, provider approached and reasons for choice of provider. ‘Care-seeking’ was defined as any action taken by a chest symptomatic to get relief for his/her symptom. The interviews were completed between March and August 2008.

Data was entered in the data star entry package. All records were entered twice to ensure accuracy. Univariate analyses were performed using SPSS version 14. Categorical variables were compared using the chi-square test. Odds ratios from logistic regression and their 95% confidence intervals were used for the interpretation of bivariate analysis. Continuous variables were compared using Mann-Whitney U test. The level of statistical significance was defined as *p*-values <0.05.

## Results

### Prevalence of chest symptomatics (Table 1)

A total of 5,708 households (rural and urban combined) comprising of 23,778 family members were contacted (not tabulated). Of these, 18,417 persons >15 years were eligible for the screening after obtaining their informed consent. The total number of chest symptomatics identified was 640 (Rural [R] 314); Urban [U] 326).

**Table 1:** Prevalence of chest symptoms by age and sex among urban and rural adults, southern India, 2008.

Age (years)	Rural						Urban					
	Males		Females		Total		Males		Females		Total	
	Total	CS n (%)	Total	CS n (%)	Total	CS n (%)	Total	CS n (%)	Total	CS n (%)	Total	CS n (%)
15–24	973	17 (1.8)	931	21 (2.2)	1904	38 (2.0)	1514	23 (1.4)	1653	20 (1.3)	3167	43 (1.4)
25–34	793	23 (3.1)	750	26 (3.3)	1543	49 (3.2)	1456	24 (1.7)	1451	26 (1.8)	2907	50 (1.7)
35–44	612	30 (4.6)	652	41 (6.7)	1264	71 (5.6)	1178	25 (2.1)	1211	31 (2.6)	2389	56 (2.3)
45–54	456	23 (5.5)	415	24 (5.3)	871	47 (5.4)	860	29 (3.4)	846	30 (3.5)	1706	59 (3.5)
55–64	255	31 (11.7)	264	22 (8.6)	519	53 (10.2)	548	41 (7.5)	547	26 (4.7)	1095	67 (6.1)
≥65	160	33 (16.7)	198	23 (14.4)	358	56 (15.6)	348	28 (8.1)	346	23 (6.6)	694	51 (7.3)
**All**	**3249**	**157 (4.9)**	**3210**	**157 (4.8)**	**6459**	**314 (4.9)**	**5904**	**170 (2.8)**	**6054**	**156 (2.6)**	**11958**	**326 (2.7)**

CS - Chest symptomatics.

The prevalence of chest symptomatics in the rural and urban areas were 4.9% and 2.7% respectively (*p*-value <0.01). Prevalence was found to increase with age (Chi-square for trend, p<0.01).

**Figure 1:**
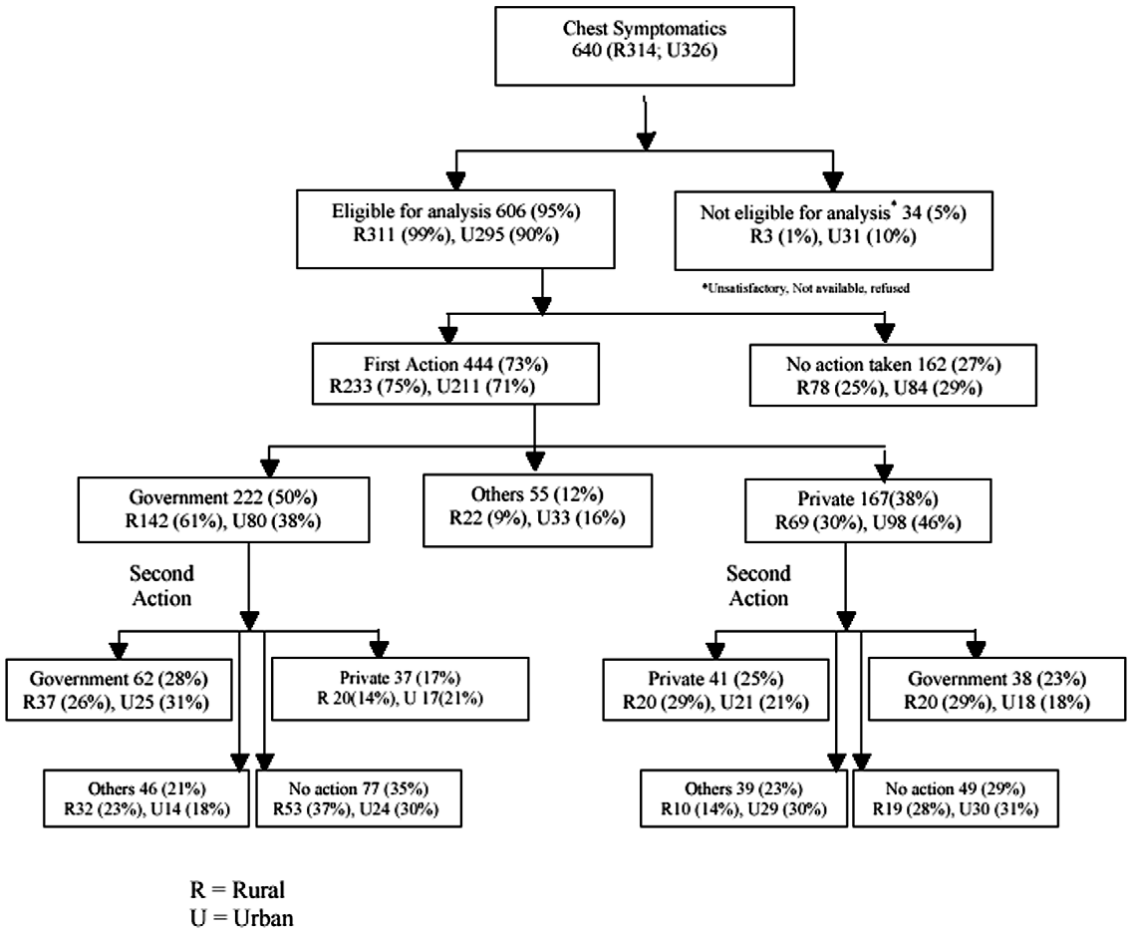
Care-seeking pattern with first provider approached (n = 640).

Detailed interviews were done for 606 (R311; U295) of the 640 symptomatics after obtaining their written consent. Thirty four (5%) could not be interviewed as 5 refused to be interviewed, 25 were not able to give satisfactory interviews, and 4 were not available in spite of three attempts made by the interviewer to contact them (Figure 1).

### Care seeking behavior of chest symptomatics

Of the 606 chest symptomatics interviewed, 444 (73%) (R75%; U71%) approached a health care provider for relief of their symptoms, and 162 (27%) (R25%; U29%) did not take any action (Figure 1). Overall, 50% of the chest symptomatics (222/444) approached a government health care facility first. Of these, 142 (61%) were rural symptomatics and 80 (38%) were urban symptomatics (*p*<0.001). Thirty eight percent (167/444), first approached a private health care facility. This included 69 (30%) rural and 98 (46%) urban symptomatic (*p*<0.001). “Others” approached included faith healers, pharmacy staff and practitioners of non-allopathic systems of medicine.

Sixty two (28%) of the 222 who went to the government facility made a second visit to the government facility (R26%; U31%), while 17% shifted to a private facility (R14%; U21%) and 21% (R23%; U18%) went to practitioners of alternate systems of medicine. Thirty five percent did not take a second action.

Of the 167(38%) who went to a private facility initially, 25% (R29%; U21%)made a repeat visit to a private facility and 23% (R29%; U18%) shifted to a government facility and 23% (R14%; U30%) went to practitioners of alternate systems of medicine. Twenty nine percent did not take second action to any facility.

### Influence of socioeconomic variables on care seeking behavior and choice of health provider (Table 2)

Age and literacy were found to be significantly associated with care seeking (p = 0.005, p = 0.000) among urban respondents. Income level was significantly associated with the choice of health care provider (p<0.05). It was found that 58.6% of those who earned ≤ Rs. 2000 per month sought care from a government health care provider while those who earned more than two thousand rupees per month sought private care.

**Table 2:** Univariate regression of socio-economic variables on first action taken to seek care for chest symptoms and their choice of service provider.

	Total	Action taken		Choice of service provider						
		*n (%)*	*OR (95% CI)*	*Government*		*Private*		*Others*		*p-value*
				*n*	*%*	*n*	*%*	*n*	*%*	
**Area**RuralUrban	311295	233 (74.9)211 (71.5)	1.19 (0.83, 1.71)1.0 Reference)	14280	60.937.9	6998	29.646.4	2233	9.415.6	<0.01
**Sex**MaleFemale	312294	225 (72.1)219 (74.5)	1.0 (Refer4ence)1.13 (0.79, 1.62)	12498	55.144.7	7790	34.241.1	2431	10.714.2	0.09
**Age (years)**<45≥45	329277	228 (69.3)216 (78.0)	1.0 (Reference)1.57 (1.09, 2.27)	106116	46.553.7	9077	39.535.6	3223	14.010.6	0.27
**Literacy**IlliterateLiterate	208398	167 (80.3)277 (69.6)	1.78 (1.19, 2.66)1.0 (Reference)	97125	58.145.1	55112	32.940.4	1540	9.014.4	0.02
**Employed**YesNo	302304	216 (71.5)228 (75.0)	1.0 (Reference)1.19 (0.83,1.71)	109113	50.549.6	7691	35.239.9	3124	14.410.5	0.37
**Income (Rs/month)** ≤2000>2000	164103	116 (70.7)77 (74.8)	1.0 (Reference)1.23 (0.70,2.14)	6830	58.639.0	3433	29.342.9	1414	12.118.2	0.03

### Reasons for shift in provider (not tabulated)

Dissatisfaction with the health services was the reason provided by 44% of respondents who had shifted from one provider to another, irrespective of the type of facility initially visited. Factors that contributed to dissatisfaction included indifference by the health care providers, delay and non-availability of health care providers when the respondent sought care. For those who shifted from government care to private, the reasons given for the shift were proximity of private providers to place of residence and the perception that “good/better care” was available in the private sector. For those who shifted from private to government care, financial constraints was the most common reason provided for the shift in care provider.

### Interval between onset of symptoms and care (Table 3)

The median interval between onset of symptoms and seeking care was uniformly 10 days in all categories of subjects, with slight differences in the range. Though the interval between onset of symptoms and care is non-normally distributed, for simplicity of interpretation, the mean and standard deviation is given. It was observed that there were longer delays among symptomatics above 45 years of age (*p*<0.05). Furthermore symptomatics who had taken previous treatment for TB, waited longer to seek care than those without a history of previous treatment.

**Table 3:** Delay in seeking care.

	Interval between onset of symptoms and seeking care (in days)		
	N	Mean ± SD	p-value
All patients	444	16±19	-
ResidenceRuralUrban	225212	16±1517±22	0.41
SexMaleFemale	220215	18±2016±19	0.33
Age<45 years≥45 years	223214	14±1819±20	0.01
LiteracyIlliterateMiddle schoolHigh school and above	16221756	18±2015±1716±25	0.49
Monthly family income (Rs.)≤2000>2000	143292	16±1816±20	0.94
Previous ATTYesNo	57378	18±1516±20	0.05

### Reasons why respondents did not seek medical care (Table 4)

The main reason provided by both rural and urban respondents for not seeking care was that their symptoms were not severe enough (R26%; U26%). Pressure of work was the second common reason (R15%; U8%), and dissatisfaction with facility (R14%; U7%). Other reasons given were financial constraints, lack of proximity and dependence on alcohol, and inconvenient working hours.

**Table 4:** Reasons given by chest symptomatics for not seeking health care.

Reasons*	Total (n = 162)		Rural (n = 78)		Urban (n = 84)	
	No.	%	No.	%	No.	%
Symptoms not severe enough	42	25.9	20	25.6	22	26.2
Pressure of work	19	11.7	12	15.4	7	8.3
Lack of money	14	8.6	7	9.0	7	8.3
Distance and lack of transport	4	2.5	3	1.2	3	3.8
Indifference	2	1.2	2	2.6	-	-
Dependence on alcohol/drugs	4	2.5	3	1.2	3	3.8
Domestic preoccupation	2	1.2	1	1.3	1	1.2
Dissatisfaction with available health facility	17	10.5	11	14.1	6	7.1
Others (specify)	23	14.2	13	16.7	10	11.9

*Multiple reasons were provided by respondents.

### Awareness of TB (not tabulated)

The most commonly reported cause of TB was germs (R35%; U26%), followed by smoking (24% in both). More than half of the urban and rural symptomatics were aware that TB is spread mainly through the air. Other modes of transmission expressed were water, food and touch. The most common source of information on TB were neighbours (R63%; U46%), TB patients (R30%; U36%,), and health providers (R25%; U15%). More than 75% of both rural and urban respondents said that TB was curable. Sixty nine percent of the rural and 62% of urban symptomatics were aware that treatment for TB was available close to their residence.

### Comparison of health seeking behavior of chest symptomatics between pre- and post-RNTCP implementation

The government facility was the first or initial point of contact for 38.4% of the chest symptomatics in the pre-RNTCP study (Table 5). This has significantly increased to 50% in the current study (p<0.001). This change is significantly more in the rural area (p<0.001), the proportions being 46.3% and 60.9% in the pre- and post-RNTCP implementation studies respectively.

**Table 5:** Comparison of health seeking between pre- and post-RNTCP implementation.

	Total					Rural					Urban				
	1^st^ Survey		2^nd^ Survey		*p*-value	1^st^ Survey		2^nd^ Survey		p-value	1^st^ Survey		2^nd^ Survey		p-value
	*n*	*%*	*n*	*%*		*n*	*%*	*n*	*%*		*n*	*%*	*n*	*%*	
**First action**	463	71.3	444	73.3	0.446	214	63.1	233	74.9	0.001	249	80.3	211	71.5	0.011
GovernmentPrivateOthers	17824540	38.452.98.6	22216755	50.037.612.4	0.0000.0000.065	9910312	46.348.15.6	1426922	60.929.69.4	0.0000.0000.127	7914228	31.757.011.2	809833	37.946.415.6	0.1640.0240.166
**Shifting of facilities**Government → PrivatePrivate → Government	4437	24.720.8	3741	16.724.6	0.0460.016	2217	22.216.5	2020	14.129.0	0.1010.143	2220	27.814.1	1718	21.318.4	0.3340.372

Similarly, the current study shows a significant decrease in the proportion seeking private facilities as first option for care, from 52.9% in the pre-RNTCP study to 37.6% in the current study (p<0.001). This significance is seen in both the rural (p<0.001) and urban areas (p<0.05), although greater in the rural area.

Considering the shift in facilities when a second care provider was sought for relief of chest symptoms, a notable finding is that there is a significant increase in the shift from private to government. The proportion that shifted from private to government facility was 20.8% in the pre-RNTCP study, while it has increased to 24.6% in the post-RNTCP study (p<0.05). There is also a decrease in the shift from government to private from 24.7% to 16.7% (*p* 0.05). This is seen in both the rural and urban areas. Pre-RNTCP 7.8% Remained with the government facility for their second action, compared with 27.9% in the current study (p<0.001). This difference is 8.1% to 26.1% in the rural area and 7.6% to 31.3% in the urban area.

## Discussion

The findings of this study on health seeking behavior of chest symptomatics after the implementation of the RNTCP have provided valuable insights on the reach of the RNTCP. The most encouraging finding is that when compared to the findings of previous studies conducted prior to the introduction of the RNTCP, there has been an overall increase of chest symptomatics seeking care for their chest symptoms in government facilities [2],[3]. It is further encouraging that in the rural areas nearly two thirds of chest symptomatics sought care initially from government health facilities. Age and literacy were significant variables that influenced care seeking behavior among urban respondents. Those above 45 years and those were not literate were more likely to seek care. This was similar to a previous study which concluded that more urban people as compared to rural areas and people in the 46–65year age group compared to younger persons sought care [4]. Another significant finding is that almost a quarter of chest symptomatics who reported to private facilities initially, subsequently shifted to a government facility for further care. These findings suggest that, because of the decentralized nature of the RNTCP service provision which is delivered keeping accessibility in mind, government facilities are now increasingly being approached. These findings are similar to another recent study on health seeking behavior of TB patients after the implementation of RNTCP [5].

Despite the establishment of decentralized TB services under RNTCP, the study observed that more than one third of chest symptomatics still sought care initially in private facilities and a quarter of them continued care in the private facility. Income seems to have influenced choice of provider in that those with higher incomes seeking private care. This is however not a negative outcome as an essential strategy is to involve private care providers in TB control. However it is worrisome that in spite of half of the respondents initially reporting to the government facilities, only a quarter subsequently remained in the government facilities for care, whilst the others shifted to private facilities or facilities offering other systems of care. Dissatisfaction with the health provider, including indifference to patients' needs, delays and non availability of the health care provider, were some of the reasons reported by the respondents. This is similar to the findings reported in other studies both in India and Bangladesh [2], [6]. As for those respondents who shifted from private facilities to government facilities, financial constraints was the main reason reported.

A matter of concern is that although the majority of chest symptomatics sought care within a few weeks of their symptoms, a significant proportion of patients either took no action or delayed seeking care. The main reason given for this delay was that symptoms were not severe enough to seek care. Another observation, as reported in other studies, was that delay increased with age [2], [7], [8]. A study in China reported that the younger age group accessed public health facilities earlier [9]. As RNTCP moves to further increase case detection by implementing more “active” case finding activities, these issues will need to be considered and addressed. Another salient finding was that symptomatics who had taken previous treatment for TB were found to wait longer before seeking care compared to those without a history of previous treatment. This weakness needs to be focused on especially as these patients will require a retreatment regimen and are also at a higher risk of having drug resistant TB.

### Limitations and strengths of the study

The limitations of this study is as seen in the previous study [2] with the concerns related to possible interviewer bias and re-call bias on the part of the respondents with regard to the type of symptoms, timeliness of care-seeking and type of health providers consulted. However as expressed already this bias was equally applicable across all respondents. In keeping with the previous study [2] in order to make comparisons the same areas with similar socio-demographic characteristics were chosen for this study. The strength of the study was that the data were obtained from a considerable number of chest symptomatics both from rural and urban settings.

### Conclusions

The RNTCP, based on a “passive” case-finding strategy appears to have had an impact in relation to the availability and accessibility of quality TB services provided through government health facilities. This is reflected in the health care seeking behavior of chest symptomatic accessing government facilities. However the findings of the current study highlight continuing challenges to the programme. There remains an urgent need to increase the involvement of private health providers in the delivery of RNTCP services, and for greater sensitization of all health care providers at all levels. In addition to decentralizing TB services and making it more accessible, it is extremely important that care is quality assured and delivered in a patient friendly manner so that patients continue and complete treatment at the government health facilities. This should include the avoidance of delays to the patient, clinic staff being available at all times, and opening hours being convenient to patients and care being more personalized as often delivered in private health care settings. There also remains a great deal to be done to increase the levels of awareness of the wider community on TB and the RNTCP services, especially in the urban settings, amongst the older age groups and those who have previously been treated for TB. However any increase in knowledge amongst the community, must go hand in hand with behaviour change towards chest symptomatics seeking timely health care free of cost.
